# *TGFBR3* Polymorphisms (rs1805110 and rs7526590) Are Associated with Laboratory Biomarkers and Clinical Manifestations in Sickle Cell Anemia

**DOI:** 10.1155/2020/8867986

**Published:** 2020-09-30

**Authors:** Rayra Pereira Santiago, Camylla Vilas Boas Figueiredo, Luciana Magalhães Fiuza, Sétondji Cocou Modeste Alexandre Yahouédéhou, Rodrigo Mota Oliveira, Milena Magalhães Aleluia, Suellen Pinheiro Carvalho, Cleverson Alves Fonseca, Valma Maria Lopes Nascimento, Larissa Carneiro Rocha, Caroline Conceição Guarda, Marilda Souza Gonçalves

**Affiliations:** ^1^Laboratório de Investigação em Genética e Hematologia Translacional, Instituto Gonçalo Moniz, FIOCRUZ-BA, Salvador 40296-710, Brazil; ^2^Universidade Federal da Bahia, UFBA, Salvador 40170-110, Brazil; ^3^Departamento de Ciências Biológicas, Universidade Estadual de Santa Cruz, UESC, Ilhéus 45662-900, Brazil; ^4^Fundação de Hematologia e Hemoterapia do Estado da Bahia, HEMOBA, Salvador 40286-240, Brazil

## Abstract

Individuals with sickle cell anemia (SCA) present chronic anemia, hemolysis, an exacerbated inflammatory response, and heterogeneous clinical complications, which may be modulated by the transforming growth factor beta (TGF-*β*) pathway. Thus, we aimed to investigate polymorphisms (*rs1805110* and *rs7526590*) of the transforming growth factor beta receptor III gene (*TGFBR3*) with regard to laboratory biomarkers and clinical manifestations in individuals with SCA. Hematological, biochemical, immunological, and genetic analyses were carried out, as well as serum endothelin-1 measurements. The minor allele (A) of the *TGFBR3 rs1805110* polymorphism was associated with increased hemoglobin, hematocrit, reticulocyte counts, total cholesterol, low-density lipoprotein, uric acid, and endothelin levels, as well as decreased platelet distribution width (PDW) and the occurrence of bone alterations. The minor allele (T) of *TGFBR3 rs7526590* was associated with increased red cell distribution width, PDW, alkaline phosphatase, aspartate aminotransferase, total and indirect bilirubin, and lactate dehydrogenase levels, as well as lower ferritin levels and the occurrence of leg ulcers. Our data suggest that the minor allele (A) of *TGFBR3 rs1805110* is associated with inflammation and bone alterations, while the minor allele (T) of *TGFBR3 rs7526590* is related to hemolysis and the occurrence of leg ulcers.

## 1. Introduction

Sickle cell anemia (SCA) is an inherited hematological disorder characterized by the presence of the beta allele S (*β*^S^) in homozygosis. SCA, the most common and severe form of sickle cell disease (SCD), is associated with more prominent hemolytic anemia, vaso-occlusive events (VOE), and a broad spectrum of clinical complications [1, 2].

Clinical manifestations in SCA are closely related to two main pathophysiological mechanisms: hemolysis and vaso-occlusion. Hemolysis reduces the bioavailability of nitric oxide (NO), which alters the homeostasis of vascular functions, triggering several clinical manifestations, such as pulmonary hypertension (PH), priapism, and stroke. Sickle erythrocyte adherence can also occur due to decreased NO bioavailability, leading to VOE, acute chest syndrome (ACS), and osteonecrosis [3–5].

In accordance with the heterogeneity and complexity of SCD, three subphenotypes have been established: viscosity vaso-occlusive, hemolysis-endothelial dysfunction, and dyslipidemic [3, 4]. The former subphenotype refers to individuals with high hemoglobin levels and an elevated frequency of clinical manifestations associated with the sickling of red blood cells (RBC), such as osteonecrosis, ACS, VOE, and pain crisis [3]. Differently, the hemolysis-endothelial dysfunction subphenotype characterizes individuals with more intense anemia and hemolysis who present clinical manifestations including stroke, leg ulcers, priapism, and PH [3, 6]. In the dyslipidemic subphenotype, individuals present an inflammatory profile decreased levels of low-density lipoproteins (LDL-C), high-density lipoprotein (HDL-C), and NO [4].

The influence of an individual's genetic background on the spectrum of clinical manifestations in SCD has prompted the search for novel biomarkers of disease severity. In this context, the transforming growth factor beta receptor III (*TGFBR3*) gene, also known as betaglycan, which is expressed in endothelial and hematopoietic cells, fibroblasts, and other cell types, is thought to be a candidate genetic factor that modulates SCA severity [7]. *TGFBR3* encodes a receptor of the transforming growth factor beta (TGF-*β*) family, TGF-*β* type III receptor (T*β*RIII), which presents affinity to all three TGF-*β* isoforms [8]. Polymorphisms in *TGFBR3* have been previously associated with clinical manifestations in SCD, such as pain crisis, ACS, infection, stroke, leg ulcers, priapism, osteonecrosis, and PH [7, 9–15]. A previous report suggested the association of *TGFBR3 rs7526590* with priapism in individuals with SCD [9]. Another polymorphism, *TGFBR3 rs1805110*, despite not being previously investigated in the context of SCD, was associated with Behcet's disease, an inflammatory disorder in the Chinese population characterized by blood vessel inflammation [16].

Carvalho et al. described lower TGF-*β* levels in individuals with SCD in crisis state in comparison to those in steady state or healthy volunteers [17]. The TGF-*β* pathway, which consists of TGF-*β*, activins, and bone morphogenetic proteins (BMP), has been implicated in a shortened life expectancy in individuals with SCD [18]. Moreover, inflammation, hematopoiesis, immune response, angiogenesis, and other cellular processes are known to be regulated by the TGF-*β* pathway [11]. Considering that these processes are also related to the pathogenesis of SCA, we endeavored to investigate polymorphisms *TGFBR3 rs1805110* and *rs7526590* with respect to associations between laboratory biomarkers and clinical manifestations in individuals with SCA.

## 2. Material and Methods

### 2.1. Subjects

The present cross-sectional study included 120 individuals with SCA, 54 (45%) females, all seen at the Bahia State Hematology and Hemotherapy Foundation (HEMOBA) from 2016 to 2017. All SCA patients were in steady state, defined as the absence of acute episodes as well as any significant medical support within the three months prior to inclusion. Median participant age was 15 years (IQR 12-17).

The research protocol was approved by the Institutional Research Board of the São Rafael Hospital (HSR protocol number 1400535) and was conducted in accordance with the Declaration of Helsinki (1964) and its subsequent revisions. All individuals or their legal guardians agreed to the biological sample collection procedures and provided signed terms of informed consent. Data pertaining to clinical manifestations was collected from patient medical records.

### 2.2. Hematological and Biochemical Parameters

Blood samples were collected by HEMOBA staff following a fasting period of no less than 12 hours. Hematological and biochemical parameter analysis was performed at the Clinical Analyses Laboratory of the College of Pharmaceutical Sciences, Federal University of Bahia (FACFAR-UFBA), while genetic and immunological analyses were performed at the Laboratory of Genetic Investigation and Translational Hematology at the Gonçalo Moniz Institute-FIOCRUZ (LIGHT-IGM/FIOCRUZ), both located in Salvador, Bahia-Brazil.

A Beckman Coulter LH 780 Hematology Analyzer (Beckman Coulter, Brea, California, USA) was used to quantify hematological parameters. Hemoglobin profiles were determined by high-performance liquid chromatography using an HPLC/Variant-II hemoglobin testing system (Bio-Rad, Hercules, California, USA).

Biochemical parameters, including lipid profile (total cholesterol and high-density lipoprotein cholesterol (HDL-C), low-density lipoprotein cholesterol (LDL-C), and triglycerides), renal profile (urea and creatinine), hepatic profile (alanine transaminase (ALT), aspartate transaminase (AST), gamma glutamyl-transferase, alkaline phosphatase, uric acid, total protein, and fractions), total bilirubin and fractions, iron, and lactate dehydrogenase (LDH) were determined using an automated A25 chemistry analyzer (Biosystems S.A., Barcelona, Catalunya, Spain). C-reactive protein (C-RP) and alpha-1 antitrypsin (AAT) levels were measured using the IMMAGE® Immunochemistry System (Beckman Coulter Inc., Pasadena, California, USA). Ferritin levels were measured using the Access 2 Immunochemistry System (Beckman Coulter Inc., Pasadena, California, USA).

Serum endothelin-1 levels were measured using an Endothelin-1 Quantikine ELISA Kit (R&D Systems, Minneapolis, MN, USA) in accordance with the manufacturer's protocol.

### 2.3. Genetic and Linkage Disequilibrium Analyses

Genomic DNA was extracted from peripheral blood using a QIAamp DNA Blood Mini Kit (QIAGEN, Hilden, Westphalia, Germany) following the manufacturer's recommendations. *TGFBR3* polymorphisms (rs1805110 and rs7526590) were detected, and genotyping was performed using TaqMan SNP Genotyping Assays (Applied Biosystems, Foster City, CA) on a 7500 Fast Real-Time PCR System (Applied Biosystems, Foster City, CA).

Linkage disequilibrium between these two SNPs was determined using the SNPStats web tool, considering *D*^2^ and *r* values. [19]

### 2.4. Statistical Analysis

All analyses were performed using the Statistical Package for the Social Sciences (SPSS) v. 20.0 software (IBM, Armonk, New York, USA) and GraphPad Prism version 6.0 (GraphPad Software, San Diego, California, USA). Significance was considered when *p* < 0.05. The Shapiro-Wilk test was used to determine the distribution of quantitative variables. The Mann–Whitney *U* test or independent *t*-test was used to compare two numerical variables depending on distribution. Hardy–Weinberg equilibrium (HWE) was assessed using the chi-squared test (*χ*^2^ test). Associations between polymorphisms and clinical data were performed using Fisher's exact test or the *χ*^2^ test with Yates correction.

## 3. Results

### 3.1. Individual Characteristics

S1 Table lists the hematological and biochemical laboratory parameters of SCA patients, with results expressed as means ± standard deviation and median (IQR).

### 3.2. Polymorphism Frequencies

With regard to the distribution of the frequencies of the *TGFBR3 rs1805110* genetic polymorphism, 71% (85/120) of the patients were homozygous for the ancestral allele (G/G), 28% (33/120) were heterozygous (G/A), and 2% (2/120) were homozygous for the minor allele (A/A) (Table 1). The analysis of *TGFBR3 rs7526590* revealed that 68% (81/120) were homozygous for the ancestral allele (A/A), 31% (37/120) were heterozygous (A/T), and 2% (2/120) were homozygous for the minor allele (T/T) (Table 1). Both polymorphisms were found to be in Hardy–Weinberg equilibrium.

### 3.3. Linkage Disequilibrium Analysis

Linkage disequilibrium (LD) calculations revealed that *rs1805110* and *rs7526590* were not in LD (*D*^2^ = 0.09; *r* = −0.0182) and, consequently, were not inherited as a haplotype.

### 3.4. Associations between Laboratory Biomarkers and Polymorphisms *TGFBR3 rs1805110* and *rs7526590*

**Figure 1:**
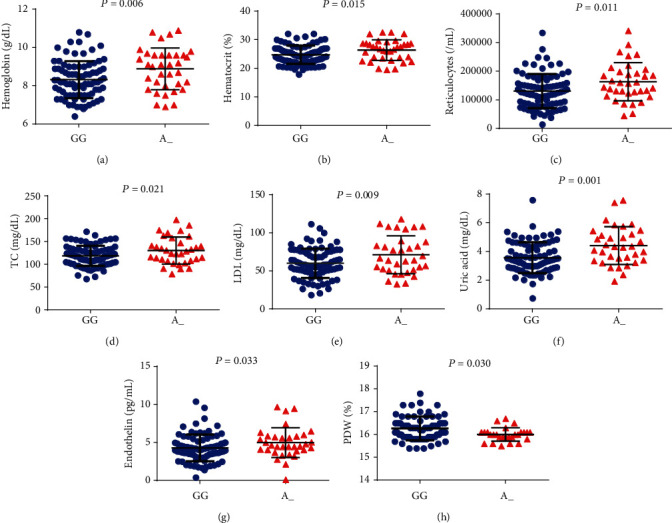
Association of *TGFBR3 rs1805110* polymorphism with laboratory biomarkers using the dominant genetic model. SCA individual carriers of the minor allele A (A/G+A/A) presented increased (a) hemoglobin, (b) hematocrit, (c) reticulocyte counts, (d) total cholesterol, (e) LDL-C, (f) uric acid, and (g) endothelin levels as well as (h) decreased PDW. All *p* values obtained by the Mann–Whitney *U* test, except for hemoglobin, hematocrit, and LDL-C, for which the independent *t*-test was used.

The dominant genetic model was employed in all analyses to evaluate associations between minor alleles and laboratory biomarkers. With regard to *TGFBR3 rs1805110*, carriers of the minor allele (A/G+A/A) presented increased hemoglobin (Hb) (*p* = 0.006), hematocrit (Ht) (*p* = 0.015), reticulocyte counts (*p* = 0.011), total cholesterol (*p* = 0.021) LDL-C (*p* = 0.009), uric acid (*p* = 0.001), and endothelin levels (*p* = 0.033), as well as decreased PDW (*p* = 0.030) (Figure 1).

**Figure 2:**
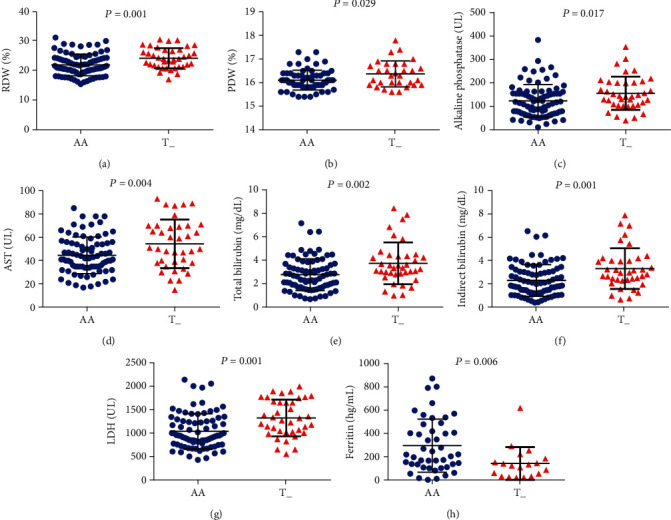
Association of *TGFBR3 rs7526590* polymorphism with laboratory biomarkers using the dominant genetic model. SCA individual carriers of the minor allele T (AT+TT) presented increased (a) RDW, (b) PDW, (c) alkaline phosphatase, (d) aspartate aminotransferase (AST), (e) total bilirubin, (f) indirect bilirubin, and (g) LDH, as well as (h) decreased ferritin levels. All *p* values obtained by the Mann–Whitney *U* test, except for RDW and AST, for which the independent *t*-test was used.

Regarding the *TGFBR3 rs7526590* polymorphism, carriers of the minor allele (AT+TT) presented increased RDW (*p* = 0.001), PDW (*p* = 0.029), alkaline phosphatase (*p* = 0.017), aspartate aminotransferase (*p* = 0.004), total bilirubin (*p* = 0.002), indirect bilirubin (*p* = 0.001), and LDH (*p* = 0.001), as well as decreased ferritin (*p* = 0.006) (Figure 2).

### 3.5. Associations between Clinical Manifestations and Polymorphisms *TGFBR3 rs1805110* and *rs7526590*

The dominant genetic model was used to investigate associations between the minor alleles of the investigated polymorphisms and clinical manifestations.

**Figure 3:**
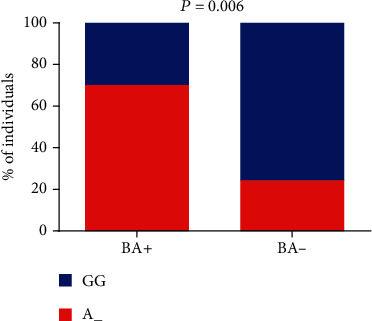
Association of *TGFBR3 rs1805110* with bone alteration occurrence (BA) among SCA individuals (*p* value was obtained by Fisher's exact test).

The minor allele (A/G+A/A) of the *TGFBR3 rs1805110* polymorphism was found to be associated with a previous history of bone alterations (*p* = 0.006) (Figure 3) (Table 2).

**Figure 4:**
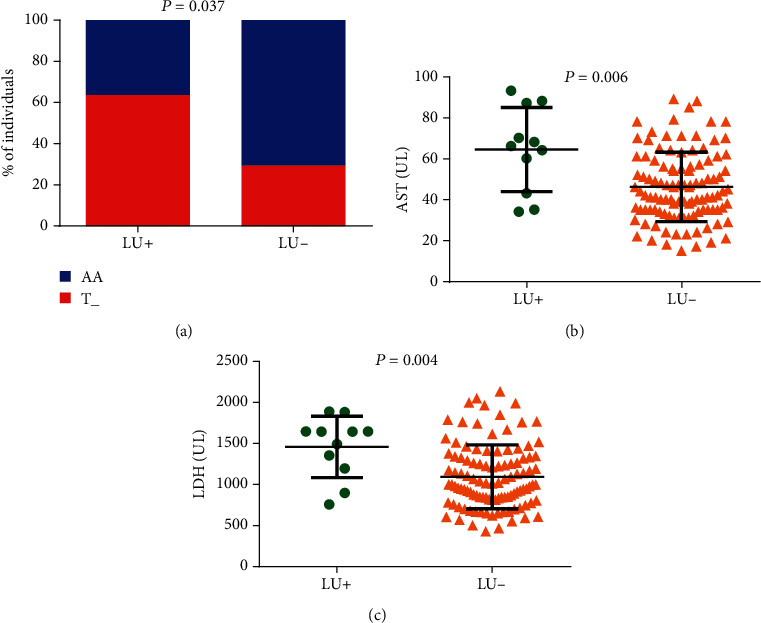
Association of TGFBR3 rs7526590 polymorphism of leg ulcer occurrence (LU). (a) SCA individual carriers of the allele T of TGFBR3 rs7526590 had high leg ulcer occurrence (*p* value was obtained with Fisher's exact test). SCA individuals with leg ulcers had high (b) AST and (c) LDH levels (*p* values were obtained by Mann–Whitney *U* test).

The minor allele of *TGFBR3 rs7526590* was associated with a history of leg ulcers (*p* = 0.037) (Table 2). We also observed that individuals with a previous history of leg ulcers (LU+) presented higher AST (*p* = 0.006) and LDH levels (*p* = 0.004) (Figure 4) than those without leg ulcers (LU-). Importantly, all individuals with a history of leg ulcers who were carriers of the T allele presented increased AST levels (*p* = 0.024).

## 4. Discussion

It has been previously described that *TGFBR3* encodes T*β*RIII, which is able to bind to all three isoforms of TGF-*β* [8]. The TGF-*β* pathway is involved in the regulation of inflammation, hematopoiesis, immune response, angiogenesis, and other cellular processes, which are relevant in the context of SCD [11]. Thus, we chose to investigate associations between the *TGFBR3 rs1805110* and *rs7526590* polymorphisms with laboratory biomarkers and clinical manifestations in a group of steady-state SCA patients.

We identified that individuals who carried the minor allele (A/G+A/A) of the *TGFBR3 rs1805110* polymorphism presented increased Hb, Ht, reticulocyte counts, LDL-C, uric acid, and endothelin levels, as well as decreased PDW. In addition, individuals with the minor allele A presented milder anemia when compared with the GG genotype. Our results suggest that inflammation was more prominent in these individuals than in carriers of the GG genotype. LDL-C plays an important proinflammatory role in vascular disease [20]. LDL-C attaches to the walls of blood vessels and becomes oxidized by reactive oxygen species (ROS), resulting in oxidized LDL. In SCA, LDL-C is more susceptible to oxidation and is considered an important marker of oxidative stress and vasculopathy [21]. Additionally, uric acid can accelerate the oxidative process in mildly oxidized LDL-C, contributing to endothelial activation and oxidative stress [22]. A previous study demonstrated the role of uric acid in activating the inflammasome pathway in individuals with SCA, leading to a proinflammatory state. These authors also demonstrated the participation of this biomarker in inflammatory events associated with SCD [23].

Carriers of the minor allele (A/G+A/A) of the *TGFBR3 rs1805110* polymorphism also had higher endothelin levels, which suggests a greater propensity of presenting vasoconstriction events and endothelial dysfunction. TGF-*β* is one of the factors that induces endothelin-1 expression by way of the overexpression of Smad3 [24]. Endothelin is a mediator produced by endothelial and immune cells, as well as neurons. This molecule acts as a vasoconstrictor by activating endothelial cells and promoting vascular inflammation [25, 26]. In addition, these individuals also presented decreased PDW, indicating better platelet uniformity than in individuals with the GG genotype [27]. An in vitro evaluation showed that TGF-*β*1 can inhibit erythropoiesis, consequently affecting thrombopoiesis, by blocking the proliferation of erythroid progenitors and accelerating differentiation in these cells [28].

The minor allele (A) of the *TGFBR3 rs1805110* polymorphism was also associated with the occurrence of bone alterations, such as osteonecrosis of the femoral head and osteomyelitis, both clinical complications commonly seen in individuals with SCD [29]. A previous study demonstrated that other polymorphisms in *TGFBR3* were associated with osteonecrosis in individuals with SCD [15]. Furthermore, the *TGFBR3 rs1805110* polymorphism was also found to be associated with Behcet's disease in both Caucasians and the Chinese Han population [16, 30]. Behcet's disease is a multisystem inflammatory disease, which can lead to the development of osteonecrosis and bone infarction in some cases [30, 31].

Our association analysis further identified high PDW in SCA patients who carried the minor allele (T) of the *TGFBR3 rs7526590* polymorphism. A previous study found that individuals with SCD presented increased PDW, suggesting bone marrow hyperplasia with the release of subfunctional platelets [27]. In addition, another study demonstrated the involvement of TGF-*β* in hematopoiesis through the control of behavior, quiescence, and the renewal of hematopoietic stem cells [32].

High levels of alkaline phosphatase, an indicator of tissue injury, were identified in SCA individuals who carried the minor allele (T) of the *TGFBR3 rs7526590* polymorphism. Previous studies have also observed high alkaline phosphatase levels in individuals with SCA [33, 34], and the literature attributes these high levels to intrahepatic sinusoidal sickling capable of damaging liver tissue [34].

The SCA individuals who carried the minor allele (T) of the *TGFBR3 rs7526590* polymorphism also presented increased RDW and higher indirect markers of intravascular hemolysis, such as AST, IB, and LDH, as well as decreased ferritin levels, which is suggestive of an association with hemolysis. During intravascular hemolysis, RBCs release LDH and AST simultaneously with hemoglobin and arginase into the bloodstream [6, 35]. In extravascular hemolysis, macrophages in the spleen and liver remove senescent and damaged RBCs, leading to the release of the heme group of hemoglobin, which is converted into unconjugated bilirubin [5]. Individuals with SCA present the most severe form of SCD due to more pronounced hemolysis have more intensive anemia and frequently present complications arising from hemolysis [1, 36].

The minor allele (T) of the *TGFBR3 rs7526590* polymorphism was found to be associated with the occurrence of leg ulcers. A recent study demonstrated that this polymorphism was associated with priapism in individuals with SCD [9]. Leg ulcers and priapism were classified by Kato et al. [3] as part of the hemolytic subphenotype, which is reinforced by Taylor et al. [37] who reported that PH, leg ulcers, and priapism were more prevalent in individuals with SCD and hyperhemolysis syndrome. Our results indicate that SCA individuals with a prior history of leg ulcers present higher levels of hemolytic markers, AST, and LDH than individuals who did not report this condition. Indeed, several studies have associated high AST and LDH levels with leg ulcers and priapism in individuals with SCD [3, 6, 38, 39]. A further analysis of the SCA individuals with a previous history of leg ulcers identified that those who carried the minor allele of the *TGFBR3 rs7526590* polymorphism presented increased AST levels. This finding serves to reinforce the association of the minor allele in this polymorphism with a hemolytic subphenotype and the occurrence of hemolysis in SCA.

Our findings suggest that *rs1805110* seems to be related to inflammation, and *rs7526590* appeared to be linked to hemolysis and clinical manifestations, especially the occurrence of leg ulcers arising from a hemolytic process. Collectively, our results corroborate the role of hemolytic parameters in addition to clinical manifestations associated with *TGBR3* polymorphism, highlighting the relevance investigating novel biomarkers of disease severity in the clinical management of individuals with SCA. To the best of our knowledge, the present study is the first attempt to demonstrate associations between these polymorphisms and hematological and biochemical parameters in SCD.

## 5. Conclusion

The results presented herein suggest that the minor allele (A) of *TGFBR3* rs1805110 is associated with an inflammatory state and the occurrence of bone alterations in SCA, while the minor allele (T) of *TGFBR3* rs7526590 is associated with a hemolytic subphenotype and related clinical manifestations, such as leg ulcers. Thus, we suggest that gene *TGFBR3* plays an important role in the physiopathology of SCA. Further studies are essential to evaluate *TGFBR3* as a prognostic marker and identify possible therapeutic targets in individuals suffering from SCD. It is important to emphasize that although this study focused on pediatric individuals, disease complications tend to worsen with increasing age.

## Figures and Tables

**Table 1:** Genotype frequencies of *TGFBR3* polymorphisms.

Individuals	Genotype frequency					
	rs1805110 (G>A)			rs7526590 (A>T)		
	G/G	G/A	A/A	A/A	A/T	T/T
Sickle cell anemia (*N* = 120)	85 (0.71)	33 (0.28)	2 (0.02)	81 (0.68)	37 (0.31)	2 (0.02)

Proportions of genotype frequency are indicated in parentheses.

**Table 2:** Association of *TGFBR3 rs1805110* and rs7526590 polymorphism with clinical manifestation in SCA.

Clinical manifestation	Polymorphisms					
	rs1805110		*p* value	rs7526590		*p* value
	GG (*N* = 85)	AG+AA (*N* = 35)		AA (*N* = 81)	AT+TT (*N* = 39)	
Acute chest syndrome	19	12	0.259	22	9	0.797
Bone alterations	3	7	**0.006** ^∗^	7	3	1.000^∗^
Cholelithiasis	27	9	0.661	26	10	0.609
Infections	60	22	0.540	52	30	0.232
Leg ulcer	9	2	0.506	4	7	**0.037** ^∗^
Pneumonia	42	24	0.086	47	19	0.444
Painful crises	48	25	0.186	53	20	0.197
Splenomegaly	34	21	0.072	38	17	0.883
Stroke	8	4	0.744^∗^	9	3	0.749^∗^
Vaso-occlusive events	28	14	0.598	28	14	0.951

Bold values indicate significance at *p* < 0.05. All *p* values obtained by the chi-squared test, except for those with asterisk (^∗^), for which the Fisher exact test was used.

## Data Availability

All relevant data used to support the findings of this study are included within the article and the supplementary information file.

## Supplementary Materials

Supplementary Materials See Supplementary Table 1 in the Supplementary Material for comprehensive data analysis.
