# Supplementary material for: TGFBR3 Polymorphisms (rs1805110 and rs7526590) Are Associated with Laboratory Biomarkers and Clinical Manifestations in Sickle Cell Anemia
Source: Dis Markers. 2020 Sep 30;2020:8867986. doi: 10.1155/2020/8867986 (PMC7547350; doi:10.1155/2020/8867986)
Supplement: Supplementary Materials — See Supplementary Table 1 in the Supplementary Material for comprehensive data analysis. [file 8867986.f1.docx]

S1 Table. Laboratory profiles of individuals with sickle cell anemia.

| **Laboratory value** | **HbSS**  **N=120** | |
| --- | --- | --- |
|  | **Mean ± SD** | **Median (IQR)** |
| Anemia and Hemolysis markers | |  |
| RBC, x10^6^/mL | 2.74 ± 0.47 | 2.65 (2.40 – 2.99) |
| Hemoglobin, g/dL | 8.48 ± 1.03 | 8.35 (7.70 – 9.17) |
| Hematocrit, % | 25.18 ± 3.39 | 25.25 (22.52 – 27.50) |
| MCV, fL | 92.64 ± 11.74 | 92.40 (86.37 – 99.15) |
| MCH, ρg | 31.41 ± 4.01 | 31.35 (29.00 – 34.05) |
| MCHC, % | 33.92 ± 1.04 | 33.85 (33.20 – 34.57) |
| Reticulocyte Count, % | 4.98 ± 1.92 | 4.80 (3.40 – 6.20) |
| Total bilirubin, mg/dL | 3.00 ± 1.56 | 2.86 (1.84 – 3.84) |
| Direct bilirubin, mg/dL | 0.41 ± 0.16 | 0.39 (0.29 – 0.52) |
| Indirect bilirubin, mg/dL | 2.59 ± 1.54 | 2.38 (1.42 – 3.40) |
| LDH, U/L | 1135.73 ± 399.98 | 1040.00 (837.50 – 1412.50) |
| Hemoglobin pattern |  |  |
| Fetal hemoglobin, % | 9.05 ± 5.68 | 7.60 (4.60 – 12.70) |
| S hemoglobin, % | 84.05 ± 9.22 | 85.70 (79.65 – 90.85) |
| Leukocytes |  |  |
| Leukocyte count, /mL | 11424.75 ± 3168.73 | 11250.00 (9125.00 – 13700.00) |
| Neutrophil count, /mL | 5522.78 ± 2425.85 | 4944.00 (3609.50 – 6906.75) |
| Eosinophil count, /mL | 398.96 ± 274.21 | 347.50 (200.00 – 593.00) |
| Lymphocyte count, /mL | 4146.18 ± 1335.15 | 4131.50 (3210.00 – 4746.00) |
| Monocyte count, /mL | 1087.63 ± 543.49 | 1100.00 (669.75 – 1404.00) |
| Platelets |  |  |
| Platelet Count, x10^3^/ mL | 421.28 ± 135.02 | 412.50 (334.50 – 498.50) |
| MPV (fL) | 7.91 ± 0.82 | 7.90 (7.32 – 8.55) |
| PDW (%) | 16.19 ± 0.48 | 16.10 (15.90 – 16.50) |
| Lipid metabolism |  |  |
| Total Cholesterol, mg/dL | 121.85 ± 24.89 | 120.00 (104.00 – 137.00) |
| HDL-C, mg/dL | 35.85 ± 8.86 | 35.00 (31.00 – 41.00) |
| LDL-C, mg/dL | 63.14 ± 21.53 | 58.80 (49.40 – 78.00) |
| VLDL-C, mg/dL | 20.31 ± 7.09 | 19.03 (14.60 – 24.05) |
| Triglycerides, mg/dL | 101.75 ± 35.27 | 96.50 (73.25 – 120.75) |
| Hepatic |  |  |
| ALT, U/L | 19.27 ± 10.09 | 17.00 (12.00 – 23.25) |
| AST, U/L | 47.81 ± 17.97 | 46.00 (35.00 – 61.00) |
| Total protein, g/dL | 8.42 ± 0.87 | 8.28 (7.85 – 9.00) |
| Albumin, g/dL | 4.79 ± 0.33 | 4.79 (4.58 – 4.98) |
| Globulin, g/dL | 3.63 ± 0.74 | 3.56 (3.12 – 4.10) |
| Alkaline phosphatase | 133.81 ± 71.35 | 127.00 (79.00 – 166.00) |
| Iron metabolism |  |  |
| Iron serum, mcg/dL | 103.28 ± 41.39 | 92.50 (75.50 – 125.50) |
| Ferritin, ηg/mL | 260.04 ± 215.27 | 178.85 (112.10 – 406.57) |
| Kidney |  |  |
| Urea, mg/dL | 17.22 ± 5.65 | 16.52 (13.98 – 20.62) |
| Creatinine, mg/dL | 0.43 ± 0.14 | 0.45 (0.32 – 0.52) |
| Inflammation |  |  |
| C-reactive protein, mg/L | 4.09 ± 2.58 | 3.34 (2.00 – 5.28) |
| Alpha 1 antitrypsin, mg/dL | 81.15 ± 44.56 | 73.85 (42.72 – 114.00) |
| Uric acid, mg/dL | 3.81 ± 1.20 | 3.58 (2.94 – 4.64) |
| Endothelin, pg/mL | 4.47 ± 1.83 | 4.34 (3.45 – 5.56) |

RBC: Red blood cells; MCV: Mean Corpuscular Volume; MCH: Mean Corpuscular Hemoglobin; MCHC: Mean Corpuscular Hemoglobin Concentration; HDL-C: High-density lipoprotein cholesterol; LDL-C: Low-density lipoprotein cholesterol; VLDL-C: Very Low-density lipoprotein cholesterol; AST: Aspartate aminotransferase; ALT: alanine aminotransferase; LDH: Lactate dehydrogenase; MPV: mean platelet volume; PDW: platelet distribution width; N: number; SD: Standard Deviation; IQR: Interquartile range.
